# Inhibition of Calpain Blocks the Phagosomal Escape of *Listeria monocytogenes*

**DOI:** 10.1371/journal.pone.0035936

**Published:** 2012-04-26

**Authors:** Gloria Lopez-Castejon, David Corbett, Marie Goldrick, Ian S. Roberts, David Brough

**Affiliations:** Faculty of Life Sciences, University of Manchester, Manchester, United Kingdom; National Jewish Health and University of Colorado School of Medicine, United States of America

## Abstract

*Listeria monocytogenes* is a Gram-positive facultative intracellular bacterium responsible for the food borne infection listeriosis, affecting principally the immunocompromised, the old, neonates and pregnant women. Following invasion *L. monocytogenes* escapes the phagosome and replicates in the cytoplasm. Phagosome escape is central to *L. monocytogenes* virulence and is required for initiating innate host-defence responses such as the secretion of the cytokine interleukin-1. Phagosome escape of *L. monocytogenes* is reported to depend upon host proteins such as γ-interferon-inducible lysosomal thiol reductase and the cystic fibrosis transmembrane conductance regulator. The host cytosolic cysteine protease calpain is required in the life cycle of numerous pathogens, and previous research reports an activation of calpain by *L. monocytogenes* infection. Thus we sought to determine whether host calpain was required for the virulence of *L. monocytogenes*. Treatment of macrophages with calpain inhibitors blocked escape of *L. monocytogenes* from the phagosome and consequently its proliferation within the cytosol. This was independent of any direct effect on the production of bacterial virulence factors or of a bactericidal effect. Furthermore, the secretion of interleukin-1β, a host cytokine whose secretion induced by *L. monocytogenes* depends upon phagosome escape, was also blocked by calpain inhibition. These data indicate that *L. monocytogenes* co-opts host calpain to facilitate its escape from the phagosome, and more generally, that calpain may represent a cellular Achilles heel exploited by pathogens.

## Introduction

*Listeria monocytogenes* is a Gram-positive intracellular bacterium that can infect a broad range of cells and is the etiological agent of the food borne infection listeriosis, affecting principally the immunocompromised, the old, neonates and pregnant women [1]. It is internalized into phagosomal vacuoles in the host cell and in order to replicate and survive must escape into the cytosol to avoid the phagolysosomal degradation machinery [1]. The two main virulence factors that facilitate escape from the phagosome are the pore forming toxin listeriolysin (LLO), encoded by the gene *hly*, and two phospholipase C enzymes, encoded by the genes *plcA* and *plcB*. *L. monocytogenes* strains that lack *hly* are non-virulent, while the virulence of strains lacking *plcA* and *plcB* is attenuated [2], [3]. Once in the cytosol *L. monocytogenes* harnesses the host cell actin polymerising machinery to facilitate motility and cell to cell spread [1], [4], [5]

Calpains are cytosolic Ca^2+^-dependent cysteine proteases that are ubiquitously distributed and comprise a family of 15 members of which μ- and m-calpain are the best characterized [6]. Calpains participate in numerous signal transduction pathways and in many important cellular processes [7]. Calpains are reported to be important for intracellular pathogen-host interactions that facilitate the life cycle of the pathogen. For example, the apicomplexan parasites *Plasmodium falciparum* (malaria) and *Toxoplasma gondii* (toxoplasmosis) co-opt host calpain to facilitate their cellular escape [8], whilst for *Cryptosporidium parvum* (cryptosporidiosis) host calpain is required for epithelial cell invasion [9]. For group B coxsackievirus (myocarditis, aseptic meningitis) infection of endothelial cells is also dependent upon host calpain [10]. Golgi mini-stack formation that occurs in *Chlamydia trachomatis* infected cells, and which is required for *C. trachomatis* maturation is also partly dependent upon calpain [11].

*L. monocytogenes* is reported to harness several aspects of the infected host cell's biology to facilitate its virulence. For example, the host enzyme γ-interferon-inducible lysosomal thiol reductase (GILT) is required for the activation of the virulence factor listeriolysin [12]. *L. monocytogenes* also exploits the cystic fibrosis transmembrane conductance regulator (CFTR) to escape from the phagosome [13]. *L. monocytogenes* is reported to induce a Ca^2+^-dependent activation of calpain in macrophages via the effects of listeriolysin [14]. The aim of this study therefore was to identify whether host calpain was important for the virulence of *L. monocytogenes* in cultured macrophages.

## Results

**Figure 1:**
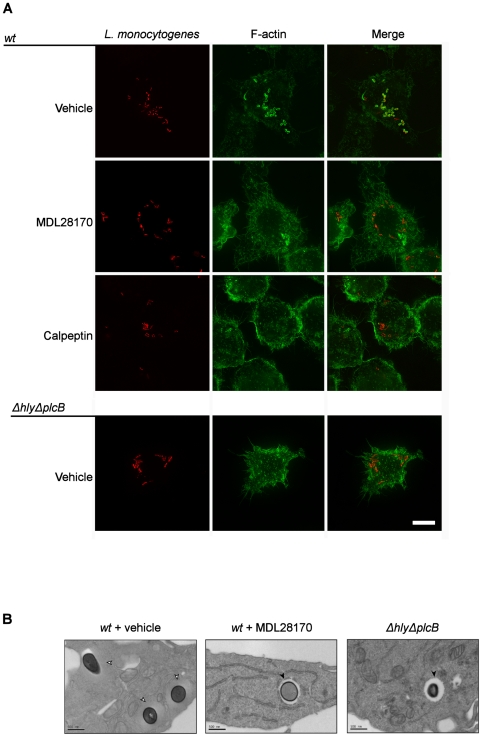
Calpain is required for phagosomal escape of *Listeria monocytogenes*. LPS-primed J774 cells pre-treated for 20 min with DMSO, MDL28170 (50 µM) or calpeptin (50 µM) were infected with *L. monocytogenes* or *ΔhlyΔplcB* expressing red fluorescent protein (RFP) strains at a MOI of 5 for 1 h and the culture was continued in the presence of gentamicin for an extra 4 h. The cells were then fixed, permeabilized and F-actin was labelled with Alexa488-phalloidin (green) (A). Transmission electron microscopy images of the same treatments (B) show bacteria free in the cytosol in vehicle treated cells (white arrow heads). In MDL28170 (50 µM) treated cells infected with wt and in cells infected with the *ΔhlyΔplcB* strain, bacteria appear in a phagosomal compartment (black arrow heads). Images representative of 2 independent experiments are shown.

**Figure 2:**
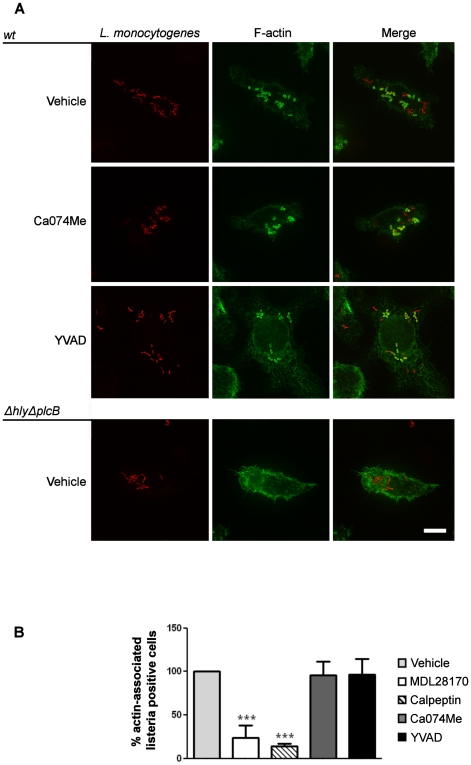
Phagosomal escape of *Listeria monocytogenes* is independent of caspase-1 and cathepsin B. LPS-primed J774 cells pre-treated for 20 min with DMSO, Ca-074-Me (50 µM) or YVAD (100 µM) were infected with *L. monocytogenes* or *ΔhlyΔplcB* expressing red fluorescent protein (RFP) strains at an MOI of 5 for 1 h and the culture was continued in the presence of gentamicin for an extra 4 h. Then cells were fixed, permeabilized and F-actin was labelled with Alexa488-phalloidin (green). Representative images of 3 independent experiments are shown (A). Percentage of infected J774 presenting actin coated *L. monocytogenes* after MDL28170 (50 µM), calpeptin (50 µM), Ca-074-Me (50 µM) or YVAD (100 µM) relative to DMSO treatment (B). Data shown as the mean ± SD, n = 3–6, ***P<0.001.

To test the hypothesis that calpain is required for phagosomal escape, mouse J774 macrophages were incubated with vehicle or the peptide calpain inhibitor MDL28170 and then infected with *L. monocytogenes* expressing red fluorescent protein (RFP). When *L. monocytogenes* is present in the cytosol the bacterial surface protein ActA activates actin polimerization [1], [15]. The detection of this polymerizing actin by phalloidin staining is indicative of phagosomal escape [16], [17]. As expected, wild type (wt) *L. monocytogenes* escaped into the cytosol of J774 macrophages and was coated in actin (Fig. 1A). In cells incubated with MDL28170, or another calpain inhibitor, calpeptin, actin coating of *L. monocytogenes* was largely absent strongly suggesting that it had not escaped the phagosome (Fig. 1A, Fig. 2B). We used the *ΔhlyΔplcB* mutant strain, which due to deletions of the virulence factors LLO and PlcB cannot escape the phagosome, as a negative control since it is known to remain trapped in the phagosome (Fig. 1A). Electron microscopy was also used to study the effect of calpain inhibition on phagosomal escape (Fig. 1B). In MDL28170 treated cells the *Listeria* were generally all found in vacuoles, again suggesting that MDL28170 blocked escape into the cytosol (Fig. 1B). In contrast the inhibitors of other cysteine proteases such as cathepsin B (Ca074Me) and caspase-1 (YVAD) had no effect on phagosomal escape (Fig. 2A, Fig. 2B).

**Figure 3:**
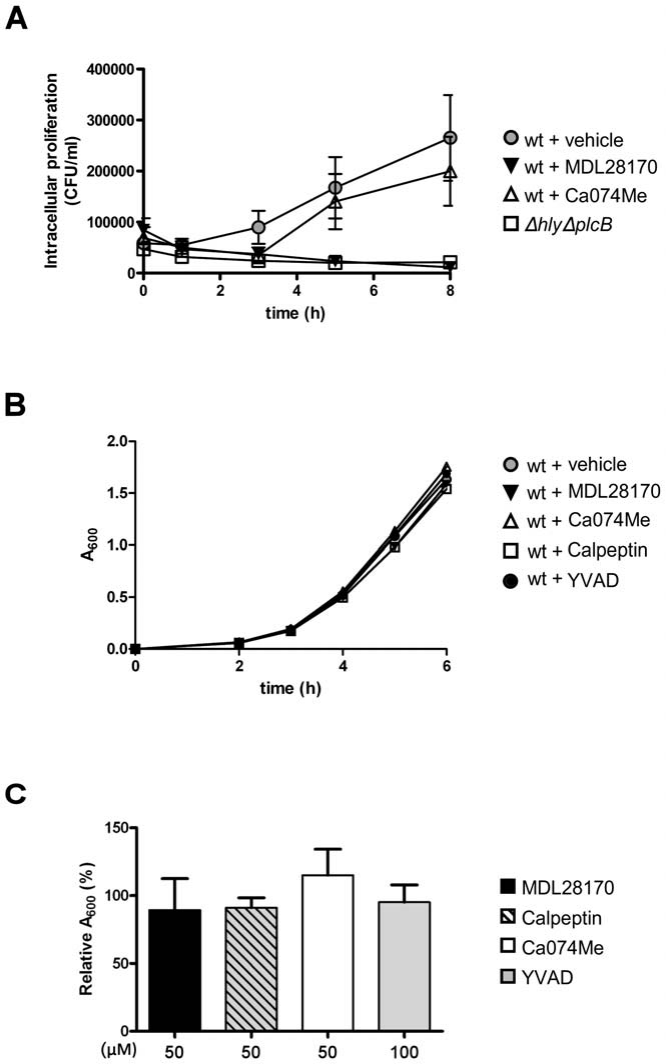
Calpain inhibition impairs intracellular growth of *L. monocytogenes*. LPS-primed J774 cells were pre-treated for 20 min with DMSO, MDL28170 (50 µM) or Ca074Me (50 µM) and infected with wt or *ΔhlyΔplcB* strains at a MOI of 1 for 1 h. After addition of gentamicin the culture was continued for another 8 h. Cells lysates were collected at different time points (0, 1, 3, 5 and 8 h) and bacterial counts obtained for each of them (A). Early effects on growth (0, 2, 3, 4, 5, 6 h) of DMSO, MDL28170 (50 µM), Ca074Me (50 µM), calpeptin (50 µM) or YVAD (100 µM) on *L. monocytogenes* expressed as A600 units (B). Effect of DMSO, MDL28170 (50 µM), Ca074Me (50 µM), calpeptin (50 µM) or YVAD (100 µM) on the growth of *L. monocytogenes* (22 h) expressed as the percentage of A600 units after inhibitor relative to vehicle treatment (C). Data showed as the media ± SEM, n = 3.

*L. monocytogenes* does not proliferate within the phagosome and requires escape for growth. Thus the interpretation that calpain inhibition blocks phagosomal escape was further supported by intracellular proliferation data showing that in MDL28170-treated, but not Ca074Me-treated, J774 macrophages growth of *L. monocytogenes* was inhibited, and was similar to the *ΔhlyΔplcB* mutant strain (Fig. 3A). It is unlikely that this effect was due to inhibition of phagocytosis *per se* since equivalent bacterial counts within the cells were observed between the MDL28170-treated wt and *ΔhlyΔplcB* infections at the initial time points after infection (0 and 60 min after gentamicin) (Fig. 3A). We then tested whether this was a direct effect of the calpain inhibitors on the viability of *L. monocytogenes* in culture. However no bactericidal or bacteriostatic effects of any of the inhibitors used in this study were observed during early phases of growth (Fig. 3B) or at 22 h when the cultures were in late exponential phase (Fig. 3C).

**Figure 4:**
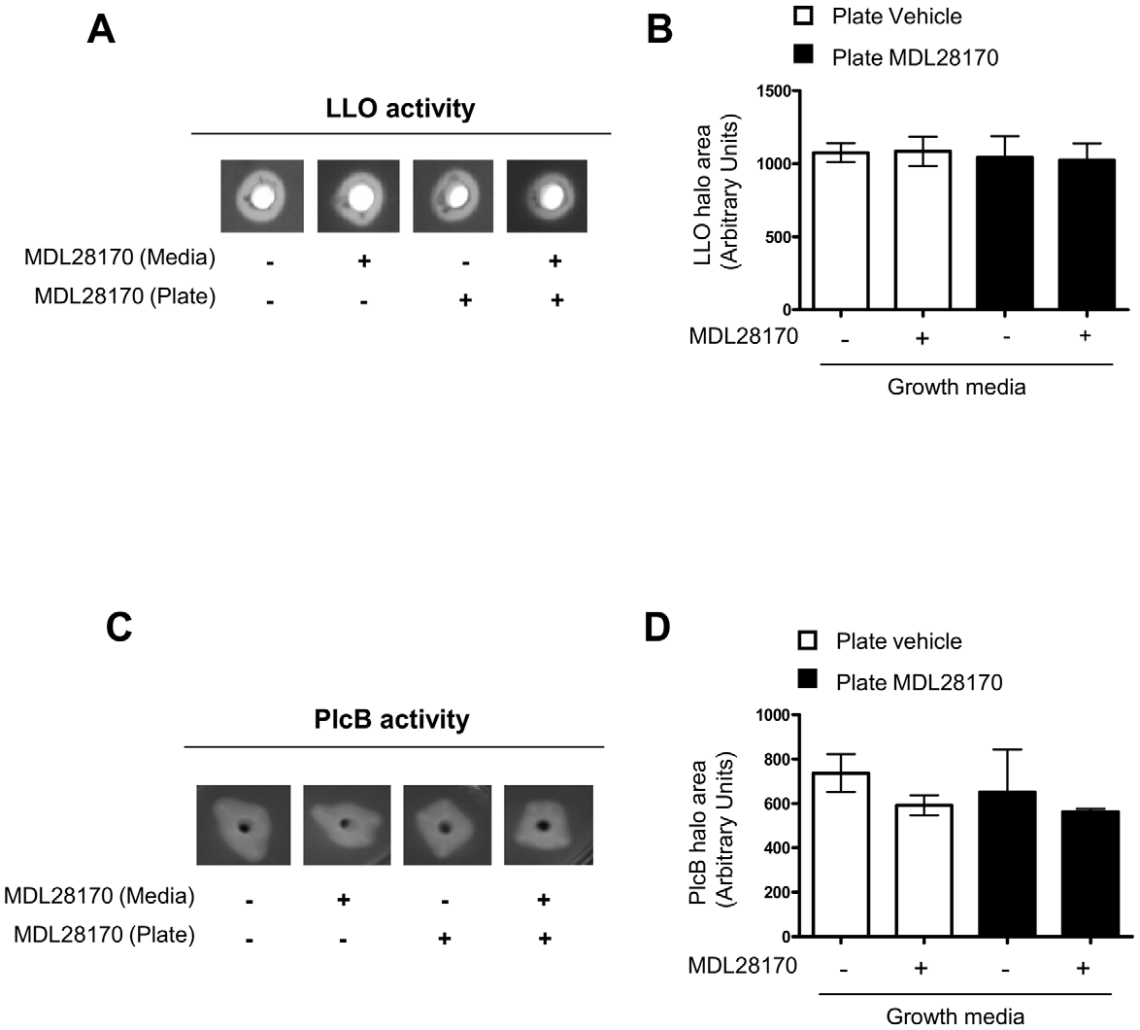
Activity of virulence factors LLO and phospholipase C are not impaired by calpain inhibitors. Cultures of C52 were grown overnight with either DMSO or 50 µM MDL28170 and bacterial supernatants were filtered. Aliquots of each supernatant were dispensed into agar plates containing defibrinated horse blood for LLO assay or 5% (v/v) egg yolk suspension for the PlcB assay in the presence or absence of 50 µM MDL28170. Plates were photographed following 36 h incubation at 37°C. LLO (A, B) or PlcB (C, D) activity was determined by the formation of a halo around the inoculation site. Halo areas were measured and plotted as arbitrary units. Data showed as the media ± SEM, n = 3 for LLO and n = 2 for PlcB activity assay.

Following internalization, *L. monocytogenes* expresses two key virulence factors that are crucial for its entry into the cytosol; the pore forming toxin listeriolysin O (LLO) encoded the gene *hly* [3] and two phospholipase C enzymes (PlcA and PlcB) encoded by the genes *plcA* and *plcB* [2]. LLO and Plc contribute to the disruption of phagosomal membrane integrity mediating escape into the cytosol. Because the effects of calpain inhibition on *L. monocytogenes*-induced phagosomal escape and intracellular proliferation were very similar to the behaviour of the *ΔhlyΔplcB* strain, we decided to study the direct effect of calpain inhibitors on the activity of LLO and PlcB. For these experiments we used the *L. monocytogenes* C52 strain that constitutively overexpresses virulence genes including *hly* and *plcB* [18]. No differences in the haemolytic activity of *L. monocytogenes* C52 were found between vehicle or MDL28170 treated bacteria as shown by the halo formation around the inoculation site (Fig. 4A, B). Similarly, the calpain inhibitor MDL28170 did not affect PlcB activity present in *L. monocytogenes* C52 (Fig. 4C, D) showing that neither LLO nor PlcB activities are inhibited by MDL28170.

**Figure 5:**
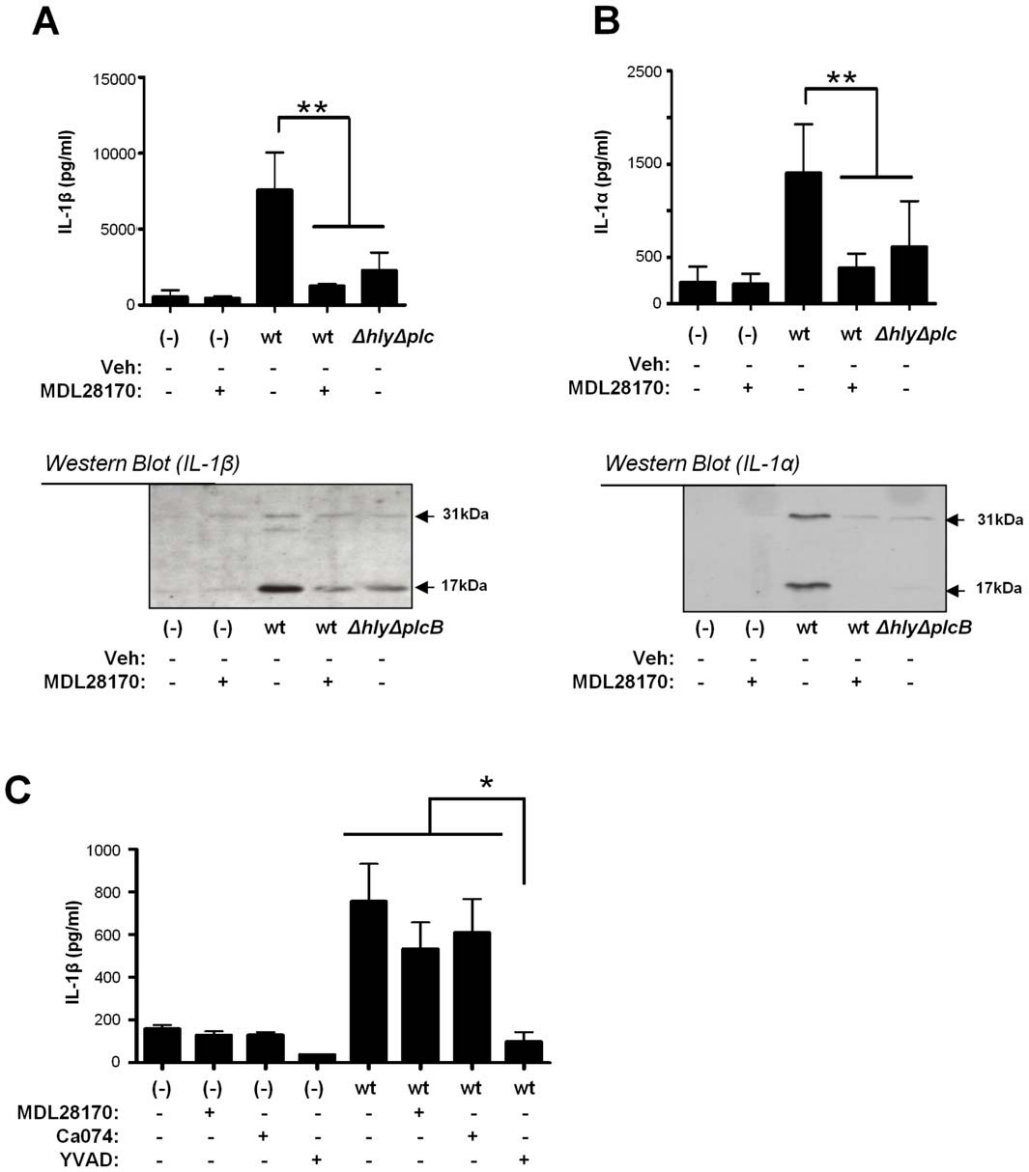
Calpain is involved in IL-1β release induced by *L. monocytogenes*, but not *S.* typhimurium infection. LPS-primed peritoneal macrophages were pre-treated for 20 min with DMSO, MDL28170 (50 µM) or Ca074Me (50 µM) before infection with wt or *ΔhlyΔplcB* strains at a MOI of 5 for 1 h. Gentamicin was then added and the culture was continued for another 3 h before supernatants were collected. Levels of IL-1β (A) or IL-1α (B) in the supernatants were measured by ELISA and western blot (precursor form 31 kDa and mature form 17 kDa). ELISA data are shown as the mean ± SD, n = 4 for MDL28170 and n = 5–7 for Ca074Me. Western blots are representative of n = 4. LPS-primed peritoneal macrophages pre-treated for 20 min with DMSO, MDL28170 (50 µM), calpeptin (50 µM), Ca074Me (50 µM) and YVAD (100 µM) and infected with *S.* typhimurium at a MOI of 30 for 1 h. After addition of gentamicin the culture was continued for another 3 h when supernatants were collected. Levels of IL-1β (C) were measured by ELISA. Data shown as the mean ± SD, n = 4–6. **P<0.01, *P<0.05.

Cytosolic pattern recognition receptors of the innate immune system sense the presence of *L. monocytogenes* in the cytosol following phagosomal escape and activate the protease caspase-1 resulting in the processing and subsequent secretion of the pro-inflammatory cytokine interleukin-1β (IL-1β) [19]. This activation of caspase-1 is essential for the clearance of *L. monocytogenes* infections [20]. IL-1β is not a substrate for calpain, although a related cytokine, IL-1α, is. *L. monocytogenes*-induced calpain activation is known to induce the processing and secretion of IL-1α, which is also dependent upon escape from the phagosome since the *Δhly* strain does not induce this activity [14]. Thus, blocking calpain activity with MDL28170 should inhibit IL-1α processing and release, but would only impair IL-1β release if phagosome escape of *L. monocytogenes* was blocked. To test this we used LPS-treated primary murine peritoneal macrophages, as opposed to J774 macrophages, since there is a robust secretion of IL-1β from these cells in response to infection, whereas J774 cells, in our hands, fail to secrete IL-1β. We found that release of both IL-1β (Fig. 5A) and IL-1α (Fig. 5B) induced by *L. monocytogenes* infection were significantly inhibited by MDL28170. This inhibition was similar to the reduction obtained following infection by the *ΔhlyΔplcB* mutant strain (Fig. 5A, B).

*Salmonella enterica* serovar Typhimurium (*Salmonella* typhimurium) is a Gram-negative bacterium that unlike *L. monocytogenes* does not escape into the cytsosol [21], [22]. However, *Salmonella* infection of macrophages still activates caspase-1 and induces IL-1β release [23]. *Salmonella* activates cytosolic pattern recognition receptors from within the phagosome through type III secretion systems and the delivery of virulence factors to the cytosol [24]. Thus we tested the effect of calpain inhibition on IL-1β secretion after infection with *Salmonella* typhimurium in LPS-treated peritoneal macrophages. The ELISA data showed that the caspase-1 inhibitor YVAD inhibited IL-1β release (Fig. 5C). However, no significant differences were found in the levels of IL-1β release when MDL28170 was used (Fig. 5C). Together these data confirm that host calpain is crucial for the escape of *L. monocytogenes* from the phagosome.

## Discussion

Here we report that inhibition of the host protease calpain blocks the release of *L. monocytogenes* from the phagosome into the cytosol. The fact that at initial time points equivalent numbers of intracellular bacteria are present across all experimental groups in the intracellular proliferation experiment (Fig. 3), and that IL-1β secretion is not affected in *Salmonella* infected macrophages treated with MDL28170 (Fig. 5), suggests that it is not phagocytosis, but truly phagosome escape that inhibition of calpain blocks.

Although not addressed in this paper it is possible to speculate as to the mechanism of calpain activation, and of its role in this system. Expression of the virulence factor LLO facilitates the escape of *L. monocytogenes* from the phagosome into the cytosol. However, preceding this event LLO perforates the phagosome membrane causing a drop in intravacuolar Ca^2+^ and its equilibration with the cytosolic compartment [25]. LLO also induces release of Ca^2+^ from the endoplasmic reticulum [26], and will therefore increase levels of cytosolic Ca^2+^ that could recruit and activate calpain [7] prior to phagosomal escape. How active calpain contributes to phagosomal escape of *L. monocytogenes* is not known. Cytoskeletal proteins constitute a major class of substrates for calpain [7], [27], and cytoskeletal protein networks constitute one of the major protein machineries driving phagosome formation [28], [29]. Disruption of the cytoskeleton can inhibit, or lead to a delay of phagosomal maturation [28]. *L. monocytogenes* induces a LLO-dependent delay in the formation of phagolysosomes that allows it to escape in to the cytosol [30]. It is possible that this delay could be explained by the recruitment of calpain and the disruption of cytoskeletal proteins. Thus it is possible that LLO-dependent calpain activation, and calpain-dependent regulation of phagosome maturation is essential for phagosomal escape. However, this requires further investigation.

It is also possible to speculate more generally about the significance of the findings reported here. The long-term treatment of, or exposure to antibiotics of bacteria has led to many strains becoming resistant, and thus poses a very significant medical problem [31]. Antivirulence drugs targeting bacterial secretion systems are emerging as a potential alternative or addition to antibiotics [32]. Such drugs avoid the intense selection pressure imposed by classical bactericidal or bacteriostatic antibiotics and are hypothesised to be less likely to lead to the development of resistance [32]. It is possible therefore that targeting host proteins co-opted by pathogens may also provide targets for antivirulence drugs. The data presented in this report, together with the literature cited in the introduction, cite calpain as a commonly exploited host cell protease in the life cycle of numerous pathogens. It may be appropriate to view members of the calpain family as an Achilles heel; the cellular weak spot subject to exploitation. Thus drugs targeting calpains may be effective in limiting the virulence of a variety of diverse pathogens including *L. monocytogenes*.

To conclude, in this study we have shown that calpain inhibition during *L. monocytogenes* infection of macrophages results in a blockade of bacterial entry into the cytosol. Furthermore, this inhibition did not occur via an inhibition of two of the principal virulence factors produced by *L. monocytogenes*, LLO and PlcB, nor was it a direct bactericidal or bacteriostatic effect. Thus we conclude that it is the activation of host calpains by *L. monocytogenes* that facilitates phagosomal escape.

## Methods

### Antibodies and reagents

Bacterial lipopolysacharide (LPS, Escherichia coli 026:B6) was purchased from Sigma. Foetal bovine serum (FBS) was obtained from PAA Laboratories. Ac-YVAD-CHO, Ca074Me, MDL28170 and calpeptin were from Merck Chemicals Ltd. The anti-mouse IL-1β and IL-1α primary antibodies used for western blot were from R&D. Secondary antibody HRP-conjugates was from DAKO. Alexa fluor 488-conjugated phalloidin was purchased from Invitrogen.

### Cells

Macrophages were prepared from adult, male C57BL/6 mice (supplied by Harlan, UK), as described previously [33]. Briefly, the peritoneal cavity was gently lavaged with RPMI 1640 media (Sigma). Cells were collected by centrifugation of the recovered media (250×g, 5 min) and plated in 24-well plates at a density of 5×10^5^ cell/well in RPMI 1640 media (Sigma) supplemented with 10% foetal calf serum (PAA Laboratories), 100units/ml penicillin and 100 µg/mL streptomycin (Sigma). The macrophages were allowed to adhere overnight (37°C, 5% CO_2_) and washed with fresh medium to remove unattached cells before use. Cultured peritoneal macrophages were LPS-primed (1 µg/ml, 2 h) to induce pro-IL-1β expression before infection.

J774 cells were cultured in DMEM (Sigma) supplemented with 10% foetal calf serum (PAA Laboratories), 100units/ml penicillin and 100 µg/ml streptomycin (Sigma). Cells were plated in 24-well plates at a density of 4×10^5^cell/well for intracellular proliferation assays and 2×10^5^cell/well for actin labelling with phalloidin. Cells were LPS-primed overnight (0.1 µg/ml) before infection.

### Bacterial strains, media and growth conditions

*L. monocytogenes* EGDe strain InlAm [34] was used throughout except during LLO or PlcB activity assays where strain C52 (NCTC7973; [35]), and phagosomal escape imaging, where *L. monocytogenes* EGDe strain InlAm transformed with plasmid pJEBAN6 which carries dsRedExpress [36], [37] were used. Glycerol stocks of *L. monocytogenes* for use in macrophage-infection experiments were prepared by culturing the bacteria in tryptone soya broth (TSB; Oxoid, UK) to A600 ∼0.6 before washing three times in an equal volume of PBS and finally being resuspended in PBS containing 10% (v/v) glycerol. *L. monocytogenes* expressing red fluorescence protein (RFP) were grown in TSB (erythromycin 20 µg/ml) and prepared in the same way. Strain *L. monocytogenes ΔhlyΔplcB* was generated in two steps. First, plasmid pLSV1-hly (Bennett and Roberts, unpublished) was transformed into *L. monocytogenes* by electroporation [37] and double-recombinants were isolated as described [38], generating *L. monocytogenes Δhly*. For the second step, primer pairs plcB-KO1F (AGATAAGAATTCTTCGTTAAGTCCAAGGTATCG) and plcB-KO1R (CGATAAGGATCCCACCTTTTTGAATTTCAT), and plcB-KO2F (ATTCTAGGATCCACAAATGAATAACAATATTTAGG) and plcB-KO2R (ATATCTAAGCTTGCGACTAACATAACTGCC) were used to generate ∼600 bp PCR fragments flanking plcB from InlAm chromosomal DNA. The fragments were then digested with the appropriate restriction enzymes (sites underlined) and ligated into plasmid pAUL-A [39]. The resulting plasmid was transformed into *L. monocytogenes Δhly*. Following plasmid integration and double recombination, only the translational start, stop and 10 codons of *plcB* remained, resulting in *L. monocytogenes ΔhlyΔplcB*. For experiments investigating the effect of inhibitors on the growth of *L. monocytogenes*, cultures were grown to early exponential phase (22 h) in a defined medium [40], or to an earlier time (6 h) in the presence or absence of inhibitor compounds, and culture densities determined spectrophotometrically (A600).

Glycerol stocks of *Salmonella enterica* serovar Typhimurium SL1344 for use in macrophage-infection experiments were prepared by culturing the bacteria in Luria-Bertani (LB) medium (1% tryptone, 1% NaCl and 0.5% yeast extract) to A600 ∼0.6 before washing three times in an equal volume of PBS and finally being resuspended in PBS containing 10% (v/v) glycerol.

### Infections

Peritoneal macrophages were pre-incubated with MDL28170 (50 µM), Ca074Me (50 µM) or YVAD (100 µM) for 20 min. After that cells were infected with *L. monocytogenes* at a multiplicity of infection (MOI) of 5 for 1 h (37°C, 5% CO_2_). For *Salmonella* infections macrophages were infected with an MOI of 30 and centrifuged for 5 min at 300×g for 1 h. After gentamicin (50 µg/ml) was added the culture was continued for 3 h.

J774 cells were pre-incubated with MDL28170 (50 µM), Ca074Me (50 µM), calpeptin (50 µM) or YVAD (100 µM) for 20 min. For actin labelling with phalloidin cells were infected with an MOI of 5 for 1 h at 37°C. Then gentamicin (50 µg/ml) was added and the culture continued for another 4 h. The cells were then washed once with PBS and fixed with paraformaldehide (4% PFA/4% sucrose). For the intracellular proliferation assays cells were infected at an MOI of 1 for 1 h. At this point some of the cells were washed twice with PBS and lysed (PBS, 5%Triton X-100). Gentamicin (50 µg/ml) was added to the other cells and the culture continued for 8 h. Cell lysates were collected as described before at 1, 3, 5 and 8 h time points.

### ELISA

Macrophage supernatants were analysed for IL-1α and IL-1β using specific ELISA kits from R&D Systems according to manufacturer's instructions.

### Western blots

Peritoneal macrophages supernatants were collected and clarified by centrifugation at 10000×g for 3 min to remove the bacteria and resolved in 15% polyacrylamide gels. Proteins were transferred to a nitrocellulose membrane and specific proteins were detected by western blotting with anti-mouse IL-1β (1∶1000) or IL-1α (1∶1000) followed by a secondary HRP-conjugated antibody, and subsequently detected using enhanced chemi-luminesence reagents (ECL, Amersham, UK).

### Intracellular growth

For the intracellular proliferation assays cells were infected at an MOI of 1 for 1 h to allow uptake of the bacteria. Cells were washed with PBS before adding DMEM media containing gentamicin (50 µg/ml). At different time points (0, 1, 3, 5 and 8 h). The media was removed and the cells washed twice with PBS. Then cells were lysed with PBS, 0.5% (v/v) Triton X-100 and the number of viable *L. monocytogenes* per well was determined by colony counting and expressed as CFU/ml.

### LLO and PlcB Activity Assays

Strain C52, that expresses a variant of PrfA resulting in constitutive overexpression of virulence genes including *hly* and *plcB* [18], was used to determine the effects of MDL28170 upon LLO and PlcB activity. Triplicate cultures of C52, InlAm, and InlAm*ΔhlyΔplcB* were grown overnight in TSB supplemented with either 0.6% DMSO or 50 µM MDL28170. Following 22 h growth, bacteria were collected by centrifugation and the supernatant passed through a 0.22 µm filter. Aliquots (80 µl) of each supernatant were dispensed into TSB agar containing either 5% (v/v) egg yolk suspension (PlcB assay) or defibrinated horse blood (LLO assay) with or without 50 µM MDL28170. Plates were photographed following 36 h incubation at 37°C. LLO or PlcB activity was determined by the formation of a halo around the inoculation site. Area of halo formation was measured using imaging software ImageJ and expressed as arbitrary units.

### Imaging and quantification of phagosomal escape

J774 cells were plated on 13 mm glass coverslips (VWR international) at a density of 2×10^5^ cells/coverslip and were infected at an MOI of 5 as described above. Cells were washed once with PBS and fixed with paraformaldehide (4% PFA/4% sucrose) for 30 min at RT. Cells were permeabilized with 0.1% triton-X 100 for 5 min at RT and actin was labelled with Alexa488-phalloidin (1∶40) in 1% BSA for 20 min. After labelling coverslips were mounted with ProLong Gold antifade. Images were acquired on a Delta Vision RT (Applied Precision) restoration microscope using a 60x/1.42 Plan Apo objective and the 360 nm/475 nm, and 555 nm/617 nm filter sets (Chroma 86000v2). The images were collected using a Coolsnap HQ (Photometrics) camera with a Z optical spacing of 0.2 µm. Raw images were then deconvolved using the Softworx software and maximum intensity projections of these deconvolved images are shown in the results.

To quantify the extent of phagosomal escape we calculated the percentage of infected cells that contained bacteria coated in actin. Cells from four different fields (average of 35 cells per field) were counted for each of the different experiments (n = 3–6) using an *Olympus BX51* upright microscope. Images were taken using a *20x/ 0.50 Plan Fln* objective and captured using a *Coolsnap EZ camera (Photometrics)* through *MetaVue Software (Molecular Devices)*. Images were analysed using ImageJ (http://rsb.info.nih.gov/ij). The data are expressed as the percentage of actin-positive cells after inhibitor relative to vehicle treatment.

For electron microscopy J774 cells were plated in at a density of 4.5×10^6^ cells primed with LPS (0.1 µg/ml) overnight before the infection. The next day LPS was removed and cells were washed once with media before pre-incubating the cells with 50 µM MDL28170 for 20 min. Cells were then infected at an MOI of 10 for 1 h when gentamicin (50 µg/ml) was added and the culture was continued for 4 h. Cells were fixed with (4% Formaldehyde, 2.5% Glutaraldehyde, 0.1 M Cacodylate buffer) and processed for transmission electron microscopy.

### Statistical analysis

Statistical analyses were performed using GraphPad Prism version 5.00 for Windows (GraphPad Software, www.graphpad.com). Differences between three or more groups were identified using one-way ANOVA with post-hoc Bonferroni multiple comparison test. Data are expressed as the mean ± standard deviation (SD) or mean ± SEM from the number of assays indicated. **P<0.01, *P<0.05.
